# The roles of advanced glycation end products in cardiovascular diseases: from mechanisms to therapeutic strategies

**DOI:** 10.3389/fcvm.2025.1637252

**Published:** 2025-09-01

**Authors:** Zhuo-Han Li, Xin-Yao Wang, Qi Luo

**Affiliations:** ^1^ Queen Mary School, Jiangxi Medical College, Nanchang University, Nanchang, China; ^2^ School of Basic Medical Sciences, Jiangxi Medical College, Nanchang University, Nanchang, China

**Keywords:** advanced glycation end products, non-enzymatic glycation, cardiovascular diseases, heart failure, therapeutic strategies, clinical translation

## Abstract

Advanced glycation end products (AGEs) are deleterious to tissues *in vivo*, arising from the process of non-enzymatic glycation (NEG), also referred to as the Maillard Reaction, which facilitates the non-enzymatic modification of biomolecules by saccharides. AGEs are integral to the physiological and pathophysiological processes associated with senescence, cardiovascular diseases (CVDs), neurodegenerative and neuroinflammatory diseases, diabetes mellitus (DM) and its complications, autoimmune and rheumatic inflammatory diseases, bone-degenerative diseases, and chronic renal diseases. Both endogenous AGEs and exogenous dietary AGEs can affect the structures and functions of proteins and lipids in cardiovascular tissues and the extracellular matrix of cardiovascular cells by inducing oxidative stress and inflammatory responses, causing direct cell and tissue dysfunction, and activating subsequent signaling pathways mediated by the AGE-RAGE axis. This review focuses on the roles and mechanisms of AGEs in CVDs, from cardiovascular tissues to concrete diseases like heart failure, valvular heart disease, and so on, together with the corresponding treatment and prevention strategies, aiming to provide a comprehensive overview of the roles of AGEs in CVDs and corresponding therapeutic measures.

## 1 Introduction

In developed nations, cardiovascular disease (CVD) is the leading cause of mortality, responsible for up to one-third of global deaths until 2015 (1, 2). The incidence of CVDs has nearly doubled, increasing from 271 million cases in 1990 to 523 million in 2019. Concurrently, mortality and disability-adjusted life years have also increased from 12.1 million to 18.6 million and from 279.8 million to 393.1 million, respectively (3). This trend is particularly pronounced in regions with low (108.3%), low-middle (114.81%), and middle (117.85%) sociodemographic indices (4). Although high-income countries have seen a decline in age-standardised cardiovascular mortality rates over the past three decades, approximately 80% of CVD-related deaths occur in low- and middle-income countries, often affecting younger individuals, with age-standardised mortality rates remaining relatively unchanged (5, 6). Additionally, the age of individuals at high risk of developing CVDs is decreasing, as evidenced by a significant rise in the global age-standardised incidence and prevalence rates of CVDs among youths and young adults from 1990 to 2019 (7). There is also a gender disparity in CVD epidemiology, with women experiencing a higher prevalence rate than men, yet having lower DALY and mortality rates (7). It is evident that CVDs pose a significant threat to global health security. Although some risk factors for CVDs, such as high systolic blood pressure, high body mass index, poor diet, elevated fasting plasma glucose, and high low-density lipoprotein levels, are well-known (6, 7), the molecular pathogenesis of CVDs remains unclear, underscoring the urgent need for further research into their pathogenesis and prevention strategies.

Advanced glycation end products (AGEs) are stable compounds formed through a complex series of non-enzymatic glycation (NEG) reactions, which begins when sugars react with proteins, lipids, or nucleic acids without enzymatic involvement, initially producing transient intermediates such as Schiff bases and Amadori products (8). This reaction was initially discovered by the French scientist Louis Camille Maillard in 1912, who termed it The Maillard Reaction. NEG can occur both inside and outside the body. In food processing, it is used to enhance flavour and texture through high-temperature cooking methods such as grilling, roasting, and deep-frying (8). Within the body, NEG is prevalent and linked to various diseases, with the accumulation of AGEs in tissues being particularly detrimental. Sullivan et al. identified AGEs and their receptor, RAGE, as a crucial factor in aging, as it causes irreversible modifications to proteins *in vivo* (9). Ahmad et al. found a connection between AGE and neurodegenerative diseases due to the accumulation of AGEs during disease progression (237). The accumulation of AGE is also associated with numerous other conditions, including neuroinflammatory diseases, diabetes mellitus (DM) and its complications, autoimmune/rheumatic inflammatory diseases, bone-degenerative diseases, and chronic kidney diseases (10).

Additionally, CVDs are another major category of illnesses attributed to the harmful effects of AGEs on cardiovascular tissues, involving atherosclerosis, hypertension, heart failure, and cardiovascular complications of diabetes, among others (11–14). For instance, Chen et al. proposed that AGEs might contribute to CVD by playing a role in atherosclerosis and arterial stiffness (15). Therefore, it is crucial to investigate the pathogenic mechanisms of NEG in CVDs, the effects of AGEs on specific cardiovascular tissues, and related therapeutic strategies.

## 2 Generation of AGEs

### 2.1 Generation process of AGEs

Advanced glycation end products (AGEs) are stable molecules generated through the non-enzymatic glycation of proteins, lipids, or nucleic acids. This process is initiated by the interaction of reducing sugars with these biomolecules, resulting in the formation of early glycation intermediates such as Schiff bases and Amadori products (16).

**Figure 1:**
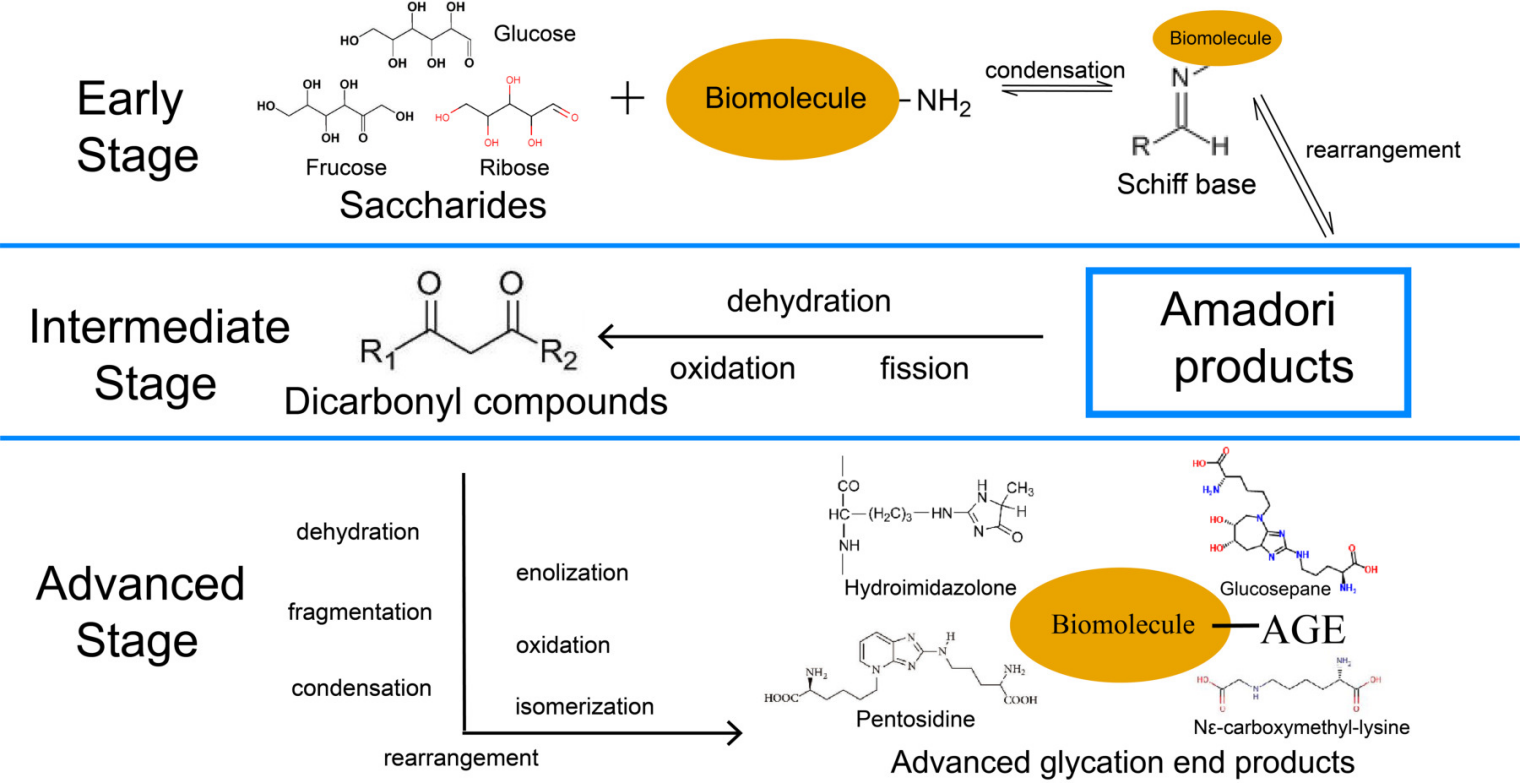
Three-stage procedure of NEG. The solid arrows represent “generation”. NEG, non-enzymatic glycation; AGE, advanced glycation end products.

According to studies, this reaction process can be roughly divided into early, intermediate, and advanced stages, with different products and characteristics at each stage (17). The carbonyl group on the open-chain form of glucose, fructose, or ribose reacts with the free amino group of biomacromolecules in the early stage with a reversible condensation reaction to form intermediate products aldimines, a type of unstable Schiff base that is susceptible to undergo rearrangement with the consequence of formation of more stable Amadori products (18, 19). In the intermediate stage, Amadori products degrade into dicarbonyl compounds through dehydration, oxidation, fission, and other reactions in an acid-base environment, containing various reactive fission products ready for further reactions (16). Finally, in the advanced stage, an extensive range of reactions, including dehydration, enolization, oxidation, fragmentation, rearrangement, isomerization, and further condensation, act on these dicarbonyl compounds, facilitating the production of irreversible AGEs, including hydroimidazolone, N*^ε^*-carboxymethyl-lysine (CML), pentosidine, and glucosepane (17, 20–22). The entire process is usually applied in food processing to control and limit critical steps (19). The overall NEG procedure is illustrated in Figure 1.

### 2.2 Factors affecting the generation of AGEs

The generation of AGEs occurs extensively in our life activities and metabolic processes, determining the functions of biological macromolecules, such as proteins, with the rate and extent of this reaction being affected by various factors within different physiological and pathological conditions.

#### 2.2.1 Blood sugar level

As the main component serving in carrying and transporting oxygen in the erythrocyte, haemoglobin (Hb) can be glycated to HbA1c under high blood sugar conditions as a measurement for monitoring blood sugar levels. Moreover, researchers have illustrated that AGE were generated in HbA1c as a precursor, can serve as a better biomarker of glycaemia in the detection of diabetic patients (23). Therefore, in the blood circulation environment, high blood sugar levels as the increased substrate and internal temperature as the appropriate temperature can enhance AGE level. Furthermore, the reaction between Hb and sugar is also found in circulation because not only the neutral *α*-amino groups from N-terminal residues of Hb has sugar molecules with high reactivity and strong affinity, the *ε*-amino groups of Lys residues in the side chain of Hb also have higher values of pH accelerating the unfolding of sugar chains and unprotonated form of the amino group, ultimately trigger the formation of AGE (17, 24). Moreover, it has been proved that the extent of reaction between proteins and carbohydrates tends to diminish with the increase of molecular weight, which means monosaccharide tend to generate more AGEs than oligosaccharide (25). Therefore, increased glucose levels in the blood are conducive to the AGE serum levels.

#### 2.2.2 Oxidative stress (OS)

The intracellular OS level significantly influences AGE production through hyperglycaemia, due to an imbalance in prooxidant-antioxidant equilibrium that favours prooxidant effects, potentially causing tissue damage (26). Research shows hyperglycaemia triggers excessive mitochondrial reactive oxygen species (ROS) production, suppressing glyceraldehyde-3 phosphate dehydrogenase (GAPDH) activity, a crucial glycolysis enzyme. This increases glyceraldehyde-3-phosphate concentration, a precursor of AGEs upstream of GAPDH, triggering AGE production (27). Oxidative stress increases AGE production and facilitates ROS generation by promoting AGE-modified proteins binding to receptors for advanced glycation end products (RAGE), a multiligand immunoglobulin superfamily member present in tissues (20, 28). Elevated AGE production under hyperglycaemia imposes various metabolic challenges in different diseases.

#### 2.2.3 Inflammatory response

Studies showed that in the myeloperoxidase system of activated neutrophils, N^ε^-(carboxymethyl) lysine (CML), a well-characterized AGE product, generates (29), indicating AGE production may increase at inflammation sites in diseases. Furthermore, binding AGEs with RAGE upregulates inflammatory cytokines, including tumour necrosis factor α, interleukin-6, and C-reactive protein, through NF-κB signalling pathways to increase inflammatory responses in CVD (30).

In addition to common pathological factors affecting NEG velocity and extent, environmental and lifestyle factors can accelerate AGE production. There are two primary AGE sources: endogenous AGEs generated in the body and exogenous AGEs ingested from foods. A high carbohydrate and calorie diet and sedentary lifestyle promote endogenous AGE production via hyperglycaemia, resulting in metabolic burdens in CVD and diabetes mellitus (DM). Exogenous AGEs form when food is processed at high temperatures, such as deep-frying, broiling, roasting, and grilling, making high-temperature cooking harmful (20). The epigenetic effects of AGEs are induced by RAGE, referring to AGEs' induction of gene expression and phenotype variation due to genetic modifications without altering DNA sequence (31). Transient alterations from short-term environmental changes in AGEs could facilitate permanent epigenetic effects (32). Long-term AGE stimulation contributes to metabolic memory, leading to adverse effects in CVD, DM, and other metabolic diseases. In DM, metabolic memory formed by increased AGE production under hyperglycaemia causes inflammatory response reinforcement, maintaining chromatin remodelling changes and histone modifications in promoters, even when blood glucose levels normalize (31). Studies indicate that increased OS and inflammatory mediators with aging are attributed to dietary AGE accumulation, having pathological effects in common diseases of older people (20, 33). Therefore, unhealthy lifestyles like sedentary behavior, high sugar diet, and high heat diet trigger endogenous and exogenous AGE accumulation, which stimulates OS and inflammatory responses, forming metabolic memory through AGEs' epigenetic effects, leading to metabolic burdens in many diseases.

### 2.3 Measurement of AGEs *In Vivo*

AGEs serve as biomarkers in CVDs and diabetes and various *in vivo* measurement methods for AGEs have been employed in clinical studies to investigate their associations with these diseases.

Due to the inherent immunogenicity of AGEs, immunochemical methods including particularly enzyme-linked immunosorbent assay (ELISA) have been widely used in clinical studies to measure AGE levels in serum or plasma (34–36). Using this approach, studies have demonstrated significantly higher serum AGE concentrations in diabetic patients compared to healthy individuals (37).

Certain AGEs exhibit spontaneous fluorescence upon excitation, enabling their quantification through fluorescence intensity measurements. This property allows the bioanalytical method for detection of AGEs in serum and urine using fluorescence spectrophotometry (FS) (38, 39), as well as noninvasive assessment of skin AGEs via skin autofluorescence spectroscopy (SAF) (40, 41). Clinical studies utilizing these methods have revealed elevated AGE levels in various disease states. For instance, Galler et al. reported significantly higher serum levels of fluorescent AGEs in type 1 diabetes patients compared to healthy controls (38), while Suehiro et al. observed increased urinary AGE concentrations in metabolic syndrome patients at high cardiovascular risk relative to healthy individuals (39). For the noninvasive way, some clinical studies have demonstrated that T2D patients had higher value of SAF compared to controls (42), and the SAF results positively correlated with coronary artery calcification and microvascular complications (43, 44). SAF was also found independently associated with all-cause mortality and fatal or nonfatal major adverse cardiovascular events in patients with peripheral artery disease (45). These findings collectively highlight the widespread application of fluorescence-based bioanalytical technologies in clinical research investigating the biomarker potential of AGEs in cardiovascular diseases and metabolic disorders.

Chromatography-mass spectrometry techniques, including high-performance liquid chromatography (HPLC), liquid chromatography-mass spectrometry (LC-MS), and gas chromatography-mass spectrometry (GC-MS), represent another analytical approach for quantifying AGEs in serum and tissue samples. However, the clinical application of these methods remains limited due to their high operational costs and time-intensive procedures (46).

## 3 Different AGE products in CVDs

### 3.1 Accumulation of AGEs in proteins

Modifications of proteins in cardiovascular tissues, including myocardial cells and vascular endothelial cells, and in plasma, will change their structures and functions, resulting in pathological effects in cardiovascular tissues.

#### 3.1.1 Structural proteins in cardiovascular tissues

Collagen and laminin are essential structural proteins in the extracellular matrix (ECM) of blood vessels and the myocardium. They are responsible for the mechanical properties of cardiovascular tissues and facilitate cell attachment, adhesion, and migration within the basement membrane (47, 48). As aging advances, there is an increased formation of AGEs on collagen and laminin within the vascular wall and myocardium. This process promotes the development of covalent crosslinks in collagen and laminin fibre, altering the matrix architecture by increasing its pore size and stiffness (47, 49, 50). Consequently, the elasticity of the ECM diminishes, resulting in decreased arterial and myocardial compliance, which increases the risk of hypertension, atherosclerosis, and cardiac failure.

Elastin is another crucial mechanical component found in the media of large and medium-sized arteries. It forms lamellae and connects the fibrils of these arteries, providing ductility (51, 52). Elastin can be viewed as a polymer composed of linear polypeptide chains (tropoelastin) stabilized by lysine-derived crosslinks (52), making it susceptible to NEG. Therefore, age-related production and accumulation of AGEs reduce elastin crosslinks, contributing to the weakening of arterial elasticity (53).

#### 3.1.2 Functional proteins in plasma

Albumin is a plasma protein that maintains osmotic pressure and transports molecules in the body. Studies show that high glycated albumin promotes mononuclear cell adhesion to endothelial monolayers for inflammatory effects (54, 55), indicating that AGEs on albumin may contribute to vascular endothelial inflammation and dysfunction in CVD. As a marker of short-term glycaemic control, glycated albumin and HbA1c are used in blood sugar monitoring (56, 57). Fibrinogen plays a vital role in forming fibrin clots and performs coagulation functions, with lysine residues being crucial (58). The level of AGEs in fibrinogen under hyperglycemia affects fibrin clot modulation and fibrinolysis, resulting in reinforced coagulation activity and resistance to fibrinolysis, increasing the propensity of fibrinogen to form clots and risk for thrombotic events in cardiovascular diseases (59–61). Therefore, fibrinogen serves as a risk marker for CVD (62) and an independent marker for vascular complications, particularly atherosclerosis (63). Myosin is a contractile protein found in cardiac and vascular smooth muscle cells (VSMC). In the context of elevated blood glucose levels, the formation of AGEs in plasma activates RAGE, which is known to engage in complex signal transduction pathways that impair the function of cardiac muscle cells and VSMCs. The mechanisms involved include an increase in overall cell rigidity through the amplification of myosin activity and a reduction in muscle contraction efficiency due to detrimental effects on the contractile capacity of the cells (64).

### 3.2 Accumulation of AGEs in lipids

In addition to the formation of AGEs in proteins, glycated lipids under hyperglycaemia or dietary levels also harm cardiovascular tissues in other ways.

#### 3.2.1 Phospholipids in cardiovascular tissues

Phospholipids are the main components of the cell membrane and are responsible for membrane fluidity. Some amino phospholipids, particularly phosphatidylethanolamine (PE) and phosphatidylserine (PS), can be glycated under conditions of hyperglycaemia or heated at high temperatures from the diet, with glycated phospholipids promoting the production of ROS and embodying angiogenic activity on endothelial cells which further exacerbates lipid peroxidation and vascular damage (65–67). Furthermore, hydrolysis of phospholipids is initiated by AGEs (68), which impairs membrane fluidity and disrupts membrane integrity and function.

#### 3.2.2 Lipoproteins in cardiovascular tissues

Lipoproteins modulate cholesterol transportation in the blood, with the two main types, low-density lipoprotein (LDL) and high-density lipoprotein (HDL), playing the roles of aggravation and resistance to atherosclerosis, respectively. It has been found that glycation of LDL will change its structure and inhibit its clearance from circulation, which provides more opportunities for its uptake by monocytes and macrophages, eliciting the generation of foam cells and the occurrence of atherosclerosis (69). Moreover, researchers have demonstrated that AGEs within both LDL and HDL under hyperglycaemia induces endothelial cell dysfunction and further atherosclerosis, with mechanisms of glycated LDL containing reduction of nitric oxide bioavailability that results in damage to the vascular system, induction of OS, decrease of fibrinolytic activity that leads to thrombus, *in vivo* atheroma formation, induction of apoptosis of endothelial cells, stimulation of inflammation like adhesion of monocytes and formation of foam cells, and activation of endoplasmic reticulum stress (70, 71). Meanwhile, AGEs impair HDL function for glycated HDL, which protects against atherosclerosis through mechanisms similar to those of glycated LDL (70).

Moreover, as the lipid composition of lipoproteins, glycated cholesterol was found to contribute to alterations in LDL clearance and increased susceptibility of LDL to OS (72), which means the enhancement of atherogenic properties of lipoproteins that are averse to atherosclerosis.

### 3.3 Accumulation of AGEs in DNA

Apart from the formation of AGEs in proteins and lipids, the glycated DNA contributes to cardiovascular tissue damage and other pathologies through mutagenesis and genomic instability.

The major nonenzymatic glycation product of DNA, N^2^-(1-carboxyethyl)-2′-deoxyguanosine (CEdG) (73, 74), has been shown to induce single-strand breaks and increase mutation frequencies in oncogenes and tumor suppressor genes (74), promote mutagenesis by altering base-pairing preferences due to its distinct syn/anti conformations during replication (75), and cause DNA damage when the nucleotide excision repair efficiency is compromised (76). These mechanisms collectively contribute to genetic instability and elevated cancer risk associated with metabolic diseases.

Acetoacetate, a ketone body increased during ketosis in diabetic patients has been found can activate effect on glucose-mediated DNA glycation during hyperglycemia because acetoacetate can induce ROS and ROS elicits DNA glycation (77). Clinical studies have found that CEdG was significantly elevated in diabetes as well, which implies the effects of DNA-AGE on diabetes and its potential on serving as biomarker of diabetes (78). Due to DNA damage resulting from many mechanisms like strand breaks induced by CEdG in the metabolic syndrome, the risk of various cancers and cardiovascular diseases will be elevated (79). However, it is worth mentioning that inhibition of glyoxylase 1 (GLO1) in cancers can increase levels of DNA-AGE and RAGE that is cytotoxic to glioma cells (80), implying the dialectical roles of CEdG in cancers. DNA damage also accompanies with aging and aggravates the incidence of neurodegenerative disease like Alzheimer disease and Parkinson disease (81).

Other structures of cardiovascular tissues impacted by AGEs are summarized in Table 1.

**Table 1:** Specific structures within cardiovascular tissues that are susceptible to glycation and their resultant effects.

Category	Substance	Function	AGE accumulation outcomes	Reference
Protein	Collagen	Mechanical properties of the cardiovascular tissues	• Increase of pore size and stiffness in matrix of vascular walls and myocardium • Reduced arterial and myocardial compliance • Induce sphingomyelinase activity, accumulation of ceramide, clustering, and later internalization of lipid rafts • Apoptosis of endothelial cells	(47, 49, 50, 82)
	Laminin	Cell attachment, adhesion and migration in basement membrane	• Increase of pore size and stiffness in matrix of vascular walls and myocardium • Reduced arterial and myocardial compliance	(47, 49, 50)
	Elastin	• Form lamellae and bridging fibrils • Ductility in arteries	Weakening of arterial elasticity	(51–53)
	Albumin	• Maintain osmotic pressure • Transport molecules	• Vascular endothelial inflammation • Endothelial dysfunction	(54, 55)
	Fibrinogen	• Form of fibrin clots • Perform coagulation function	Thrombotic events	(58–61)
	Myosin	Contractile protein in cardiac muscle cell and vascular smooth muscle cell (VSMC)	• Enhancement of overall cell rigidity • Impairment of muscle contraction	(64)
	Troponin	Facilitate cardiomyocyte contraction	• Alteration of calcium sensitivity • Impairment of cardiac contractility	(83)
	Insulin Receptor	Mediate insulin signaling in cardiovascular tissues	• Insulin resistance in diabetes • Metabolic and cardiovascular dysfunction	(84)
Lipid	Phospholipids	Cell membrane fluidity	• Peroxidation of lipid and vascular damage • Hydrolysis of phospholipids and disruption of membrane fluidity integrity	(65–68)
	Low-density lipoprotein (LDL)	Transportation of cholesterol in blood	• Endothelial cell dysfunction • Atherosclerosis	(69–71)
	High-density lipoprotein (HDL)			(70)
	Cholesterol	—	• Alterations of LDL clearance • Increased susceptibility of OS for LDL	(72)
DNA	–	• Induce single-strand breaks • Genetic instability	• Development of diabetes, cancer, neurodegenerative diseases, and aging-related dysfunction	(73–81)

## 4 The mechanisms of AGE accumulation in CVDs

The mechanism of AGE deposition in CVDs have been investigated. It has been shown that the harmful effects of AGEs in CVDs depend on their interaction with receptors, which can be categorized as either intracellular or extracellular. Intracellular AGE receptors include RAGE, a type I cell surface receptor that is part of the immunoglobulin (Ig) superfamily. This group also includes scavenger receptors such as class H, class A, class B, and class E, as well as the AGE receptor complex (AGE-R) comprising OST-48, 80K-H, and galectin-3 (85). The soluble receptor for advanced glycation end products (sRAGE) is an extracellular receptor for AGEs that exists freely outside the cells. It has two main forms: endogenous secretory RAGE (esRAGE), a splicing variant of the RAGE gene, and cleaved RAGE (cRAGE), which results from the breakdown of membrane RAGE (86). Among the various AGE receptors, the RAGE and AGE-RAGE axis has been extensively studied, allowing for a detailed exploration of the mechanisms by which AGEs act in CVDs.

### 4.1 OS and activation of inflammatory reaction

OS is linked to the levels of ROS, which include superoxide anion radicals (O^2−^), hydrogen peroxide (H_2_O_2_), and hydroxyl radicals (HO^−^). This is sometimes accompanied by reactive nitrogen species (RNS), such as nitric oxide (NO^−^), nitrogen dioxide (NO^2^), and peroxynitrite (ONOO^−^). Several mechanisms through which AGEs lead to OS and inflammation have been identified. First, AGEs create irreversible crosslinks with proteins and lipids, altering their structure and function, promoting ROS production and OS, as discussed in Sections 3.1, 3.2. Second, AGEs cause mitochondrial dysfunction by disrupting the respiratory chain and increasing mitochondrial stress, leading to excessive ROS production and OS (87). Third, AGEs cause OS and ROS production by interacting with RAGE. AGE-RAGE interaction activates nicotinamide adenine dinucleotide phosphate (NADPH) oxidase (NOX), resulting in ROS production (88, 89). Excessive ROS generation leads to abnormal oxidation and dysfunction of biomacromolecules, triggering the expression of inflammatory mediators, such as tumour necrosis factor-alpha (TNF-α), interleukin-1 beta (IL-1β), cyclooxygenase 2 (COX-2), and prostaglandin E2 (PGE2) (88). Fourth, signalling pathways activated by the AGE-RAGE axis induce OS and inflammatory responses. The nuclear factor kappa B (NF-κB) signalling pathway is the main pro-inflammatory pathway in ROS and OS production, leading to vascular injury. NF-κB is a transcription factor sensitive to free radicals that regulates the transcription of endothelin-1, VCAM-1, tissue factor, and thrombomodulin, which relate to vascular functions (90). ROS production in endothelial cells stimulated by AGEs is mediated by NF-κB signaling, where NF-κB induces TNF-α expression, mediating ROS generation (91). Additionally, Rho/Rho-associated protein kinase (Rho/ROCK) expression mediated by AGEs is involved in NF-κB signalling pathway and ROS production (92, 93). The Janus kinase-signal transducers and activators of transcription (JAK-STAT) signalling pathway is activated by AGE-RAGE, leading to JAK phosphorylation and STATs activation. Phosphorylated STATs form dimers and move into the nucleus to regulate transcription of inflammatory cytokines, such as interleukin-6 (IL-6), TNF-α, and MCP-1, and inducible enzymes like iNOS and COX2 (94). Moreover, RNS production is mediated by the AGE-RAGE axis, contributing to OS (95).

After OS and inflammatory reactions induced by AGEs, further increased production of AGEs can be triggered by OS and inflammatory reactions themselves, as mentioned in Sections 2.2.2, 2.2.3. This is how the vicious circle is generated, which gradually aggravates the implications of OS and inflammatory reactions. Furthermore, it was proven that the enhancement of NOX activity and activation of NF-κB induced by AGE-RAGE interactions will facilitate the expression and production of iNOS and ONOO^−^, namely the overproduction of RNS that also results in OS (95). RNS is involved in the production of AGEs (96), which also serves as a part of the vicious circle.

The occurrence of OS and accumulation of inflammatory cytokines such as C-reactive protein (CRP), IL-1β, IL-6, TNF-α and so on will subsequently trigger the oxidative damage of biomacromolecules *in vivo* and aggregation of inflammatory cells, which ultimately leads to the impairment of cardiovascular structures and development of CVDs.

### 4.2 Cell dysfunction

AGE can directly initiate injury in cardiovascular cells involving myocardial cells, vascular endothelial cells, smooth muscle cells, and so on to affect their normal physiological functions. Endogenous AGEs produced inside myocardial cells and cardiac fibroblasts are proven to embody cytotoxic and cause CVDs. It was indicated that AGEs in cardiomyocytes could decrease pulse rate and cell activity and induce cell death, which concerns the suppression of autophagy. It is an essential mechanism for maintaining cell homeostasis via the degradation of aging and damaged proteins and organelles (97). AGEs in cardiac fibroblasts exert cytotoxicity and inhibit their roles in regulating myocardial ECM homeostasis and restoring damaged cardiac functions. Furthermore, with the expression of RAGE in cardiomyocytes and cardiac fibroblasts, the AGE-RAGE axis may also induce cytotoxic reactions and dysfunction in these cells (98). Extracellular AGEs also facilitate CVDs through the AGE-RAGE axis, which activates the production of ROS and, subsequently, the NF-κB signalling pathway in vascular wall cells, contributing to atherosclerosis (99).

Recent studies have suggested that the interaction between AGEs and RAGE can destroy the balance of osteogenesis and osteoclastic in VSMCs to promote vascular calcification and pathological bone remodelling (100, 101), contributing to atherosclerosis development. Another mechanism that elicits calcium deposition in VSMCs is Nox-derived OS, which is involved in AGE-induced apoptosis of VSMCs (102). Moreover, it was proven that the interaction between AGEs and RAGE could induce the expression of the pro-sclerotic cytokine TGF-β, which mediates the trans-differentiation of smooth muscle cells to myofibroblasts (103), which may promote vascular stiffness through the destruction of VSMCs.

Studies have demonstrated that AGEs boost vascular endothelial growth factor (VEGF) production and activate its autocrine function in endothelial cells, resulting in angiogenesis. Overexpression of RAGE can trigger reactions in endothelial cells, highlighting the AGE-RAGE axis (104). Profilin-1, an intracellular protein associated with actin, regulates endothelial cell contraction and vascular permeability. AGEs elevate profilin-1 levels in endothelial cells, causing cytoskeletal changes and cell damage, evident from increased endothelial cell permeability, apoptosis initiation, and enhanced cell migration, adhesion, and focal contact formation (105). Additionally, myosin light chain (MLC) phosphorylation by MLC kinase (MLCK) promotes cytoskeletal contractile activity and disrupts tight junctions, increasing endothelial permeability. AGEs in endothelial cells can decrease miR-1-3p expression, a microRNA with therapeutic potential in treating endothelial dysfunction. This decrease amplifies MLCK signalling, leading to cytoskeletal contraction, increased vascular permeability, endothelial cell aging, and barrier dysfunction (106). Endothelial progenitor cells (EPCs), particularly late EPCs, vital for endothelial maintenance, repair, and postnatal angiogenesis, are negatively impacted by AGEs in a concentration-dependent manner, disrupting proliferation, migration, adhesion, and inducing apoptosis (107).

### 4.3 Extracellular matrix remodeling

In addition to direct impairment of cardiovascular cells by AGEs, modification of ECM components by AGEs leads to alterations in structure and function that contribute to CVD pathological effects. As mentioned in Section 3.1, AGEs modify essential ECM proteins, such as collagen and elastin, affecting their physiological functions by changing crosslink structures. Ligation of AGEs and RAGE may alter the production of these ECM proteins, leading to changes in elastic fibres and increased carotid diameter (108). This is associated with arterial stiffness and atherosclerosis risk. Furthermore, studies show that exercise training can inhibit advanced glycation by reducing CML and RAGE expression, which attenuates aortic stiffening and prevents endothelial dysfunction marked by decreased collagen levels, increased elastin levels, and reduced pulse wave velocity (PWV), with RAGE inhibitor FPS-ZM1 achieving similar effects (109). This indicates the dynamic relationship between collagen and elastin relates to vascular physiological conditions. AGEs may attenuate elastin levels and increase collagen levels to aggravate aortic stiffness, contributing to CVD pathological responses. Beyond glycated collagen that can attenuate vascular wall flexibility and arterial and myocardial compliance, as noted in Section 3.1.1, glycated structural extracellular proteins contribute to myocardial stiffness, causing impaired relaxation and diastolic dysfunction (110). Glycated type IV collagen and laminin can reduce endothelial cell adhesion and spreading by disrupting cell attachment sites (111), destroying endothelial cell integrity and functions.

Glycation of other ECM components, such as fibrinogen and LDL, as mentioned in Section 3.1.2, generally contributes to thrombogenesis, reduction of fibrinolysis, and risk of atherosclerosis. Other studies have shown that irreversible AGE-induced modifications of ECM components, such as type IV collagen, laminin, and vitronectin, can facilitate the overproduction and accumulation of ECM and vasoconstriction (112), which contributes to the accumulation of fibrous tissue, resulting in myocardial fibrosis and hypertension. Furthermore, the AGEs–RAGE axis induces autophagy, which can activate cardiac fibroblasts, which are responsible for the secretion of collagenous extracellular matrix proteins, such as collagen I and collagen III. Activated cardiac fibroblasts transform into myofibroblasts and accumulate in the myocardium. Increased ECM component deposition and myofibroblast accumulation contribute to myocardial fibrosis in heart failure (113). AGEs also increase the expression of KCa3.1 channels in a RAGE-dependent manner by activation of ERK1/2, p38-MAPK, and PI3K/Akt signalling, thereby promoting the proliferation of cardiac fibroblasts and ECM (114), which is another mechanism of deposition of ECM components that correlates to myocardial fibrosis. The small GTPase Rap1a modifies and activates downstream events of the AGE/RAGE signalling cascade to increase cardiac fibroblast collagen gel contraction and ECM remodelling (115). TGF-β signalling interacts with RAGE signalling or independently causes ECM remodelling (116). Consequently, myocardial fibrosis is exacerbated.

### 4.4 Effects on CVDs-related signaling pathways

**Figure 2:**
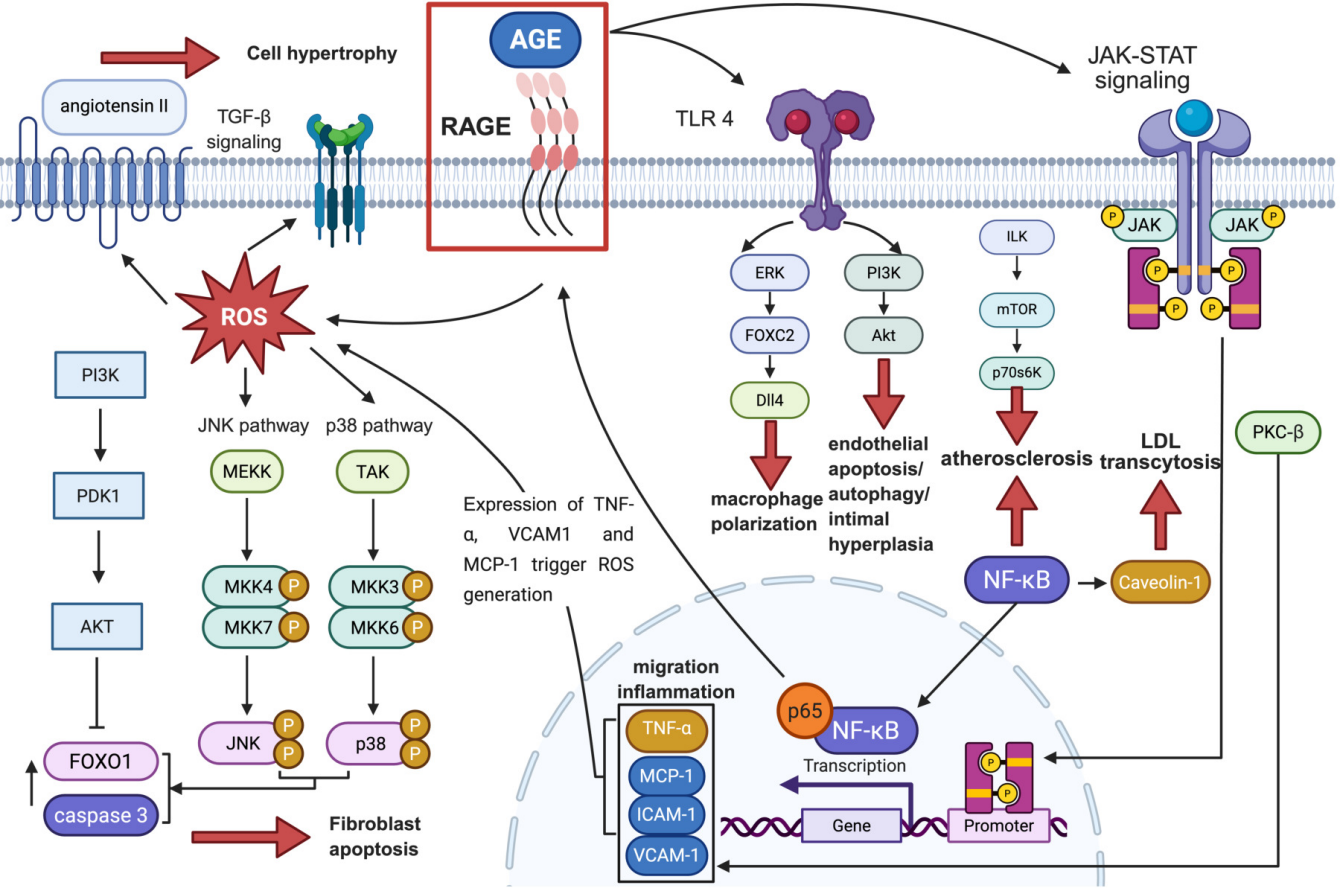
The signaling pathways associated with CVD that are mediated by the AGE-RAGE axis, along with their downstream factors and ultimate effects on pathological conditions. The black or red solid arrows represent “facilitation”, the dashed arrows represent “combination”, and the solid “T” lines represent “inhibition”. OS, oxidative stress; AGE, advanced glycation end products; RhoA, Ras homolog family member A; ROS, reactive oxygen species; Ang II, angiotensin II; TGF-β, transforming growth factor-β; Smad, suppressor of mothers against decapentaplegic; PKC-β, protein kinase C-β; MCP-1, monocyte chemotactic protein-1; ICAM-1, intercellular adhesion molecule-1; fVCAM-1, vascular cell adhesion molecule-1; JNK, c-Jun N-terminal kinase; FOXO1, forkhead box protein O1; TNF-α, tumor necrosis factor-α; NF-κB, nuclear factor kappa-B; NADPH, triphosphopyridine nucleotide; LDL, low-density lipoprotein; TLR4, toll-like receptor 4; ERK, extracellular regulated protein kinases; FOXC2, forkhead box C2; DII4, delta like ligand 4; PI3K, phosphoinositide 3-kinase; AKT, protein kinase B; ERK1/2, extracellular regulated protein kinases 1/2; JAK, Janus kinase; STAT, signal transducer and activator of transcription; ILK, integrin linked kinase; mTOR, mammalian target of rapamycin; p70S6K, p70 ribosomal protein S6 kinase.

After discussing the direct effects of AGEs on OS, inflammation, cell dysfunction, and ECM remodelling that elicit pathological responses in CVDs, we will illustrate the mechanisms by which glycation disrupts normal transduction of crucial signalling pathways in CVDs to microscopically explore the impairment of AGEs on cell metabolism and survival in the development of CVDs. A related illustration is shown in Figure 2.

Activation of the RAGE/NF-κB signalling pathway by AGEs is a prevalent mechanism that contributes to the progression of cardiovascular diseases. Recent research has shown that AGEs trigger the RAGE-NF-κB signalling cascade, leading to increased NF-κB signalling. Concurrently, the NF-κB subunit p65 is drawn to the RAGE promoter, which boosts RAGE transcription and establishes a positive feedback loop between NF-κB signalling and RAGE expression, further amplifying the NF-κB signalling pathway. Consequently, various downstream effectors of this pathway are activated to perform their respective roles. Caveolin-1, a key membrane-bound scaffolding protein found in caveolae, acts as a downstream effector of the RAGE/NF-κB signalling pathway and is involved in LDL transcytosis in endothelial cells. Enhanced NF-κB signalling leads to the upregulation of Caveolin-1, facilitating LDL transcytosis and causing LDL to deposit beneath the endothelium, which ultimately contributes to the development of atherosclerotic plaques (117).

Protein kinase C (PKC) is a collection of enzymes within the AGC family (cAMP-dependent protein kinase/protein kinase G/protein kinase C) that function as serine/threonine-related protein kinases and play a role in cell signalling (118). The RAGE/PKC-β pathway is another signaling mechanism implicated in vascular dysfunction, where AGEs trigger the increased production of monocyte chemotactic protein-1 (MCP-1) in monocytes and endothelial cells, as well as intercellular adhesion molecule-1 (ICAM-1) and vascular cell adhesion molecule-1 (VCAM-1) in endothelial cells during inflammatory conditions and oxidative stress. This process leads to macrophage adhesion to endothelial cells, contributing to endothelial dysfunction (119, 120). Additionally, AGEs influence the RAGE/PKC-β signalling pathway by enhancing the expression and phosphorylation of PKC-β1/2 in cardiac microvascular endothelial cells (CMECs), promoting CMEC proliferation and reducing early apoptosis, potentially playing a role in the development of cardiomyopathy (121).

Research shows that the RAGE/phosphatidylinositol 3-kinase (PI3K)/AKT signalling pathway is activated by AGEs and regulates cellular functions. AGEs increase the expression of proteins related to cell proliferation and migration, as well as filamentous actin, in human aortic vascular smooth muscle cells (HASMCs). This leads to HASMC proliferation and migration, causing intimal hyperplasia and lumen stenosis, conditions linked to arteriosclerosis development (122). The mechanism behind VSMC proliferation involves autophagy triggered by AGEs through the ERK signaling pathway, a member of the mitogen-activated protein kinase (MAPK) family, and AKT signaling pathways. AGEs promote ERK phosphorylation while inhibiting AKT phosphorylation (123). Studies suggest that inhibiting AGE-induced apoptosis in endothelial cells can be achieved by activating the ERK1/2 and PI3K/AKT signalling pathways (124). This indicates that AGEs' impact on apoptosis and endothelial cell death may involve the ERK1/2 and PI3K/AKT pathways.

Rho is an essential small GTP-binding protein that functions as a molecular switch in signal transduction and plays a crucial role in remodelling the actin cytoskeleton across various cell types. Research has shown that the RAGE/Rho signalling pathway can be triggered by AGEs, leading to the expression of RhoA in endothelial cells. This process enhances microvascular hyperpermeability through actin polymerization and the reorganization of the actin cytoskeleton (125).

Research has demonstrated that the interaction between AGEs and the receptor for advanced glycation end-products (RAGE) can trigger Toll-like receptor 4 (TLR4) signalling. This activation promotes ERK phosphorylation, which in turn increases the expression of FOXC2 and subsequently delta-like ligand 4 (Dll4) in macrophages during M1 polarization. FOXC2, a transcription factor from the forkhead family, is essential for vascular development and integrity, whereas Dll4 can activate the Notch signalling pathway, which is vital for the phenotypic conversion of vascular smooth muscle cells (VSMCs) from a contractile to a synthetic state. Consequently, within the RAGE/TLR4/FOXC2 signalling pathway, the high expression of Dll4 induced by AGEs in macrophages through direct cell-to-cell interaction mediates the Notch signalling pathway, leading to the formation and progression of atherosclerotic plaques characterized by VSMCs transitioning from contractile to synthetic phenotypes (126). The RAGE/ILK/mTOR/p70S6K signalling pathway is another key player in the advancement of atherosclerosis, with ILK acting as a versatile intracellular serine/threonine kinase that contributes to the pathogenesis of the disease. Within this pathway, AGE activation triggers the expression of proteins in the ILK/mTOR/p70S6K signalling pathway, leading to VSMC-to-osteoblast trans-differentiation and cell proliferation, which in turn fosters the progression of atherosclerosis (127).

Certain signalling pathways are triggered by ROS, which is facilitated by AGEs. AGE-RAGE-mediated production of ROS activates the Ang II-TGF-β–Smad pathway. In this pathway, ROS-induced autocrine production of Ang II in mesangial cells leads to TGF-β expression and Smad phosphorylation, resulting in mesangial cell hypertrophy and fibronectin synthesis (128). ROS production through AGE-RAGE interactions stimulates p38 and JNK within the MAPK signalling pathway, initiating cascades that activate cascade-3 and FOXO1, promoting fibroblast apoptosis (129). AGEs may also induce vascular adventitial fibroblasts (AFs) migration and inflammatory mediator release by upregulating RAGE expression via MAPK signalling and NF-κB activation, contributing to early atherosclerosis development (130).

## 5 The role of AGEs in different CVDs

**Figure 3:**
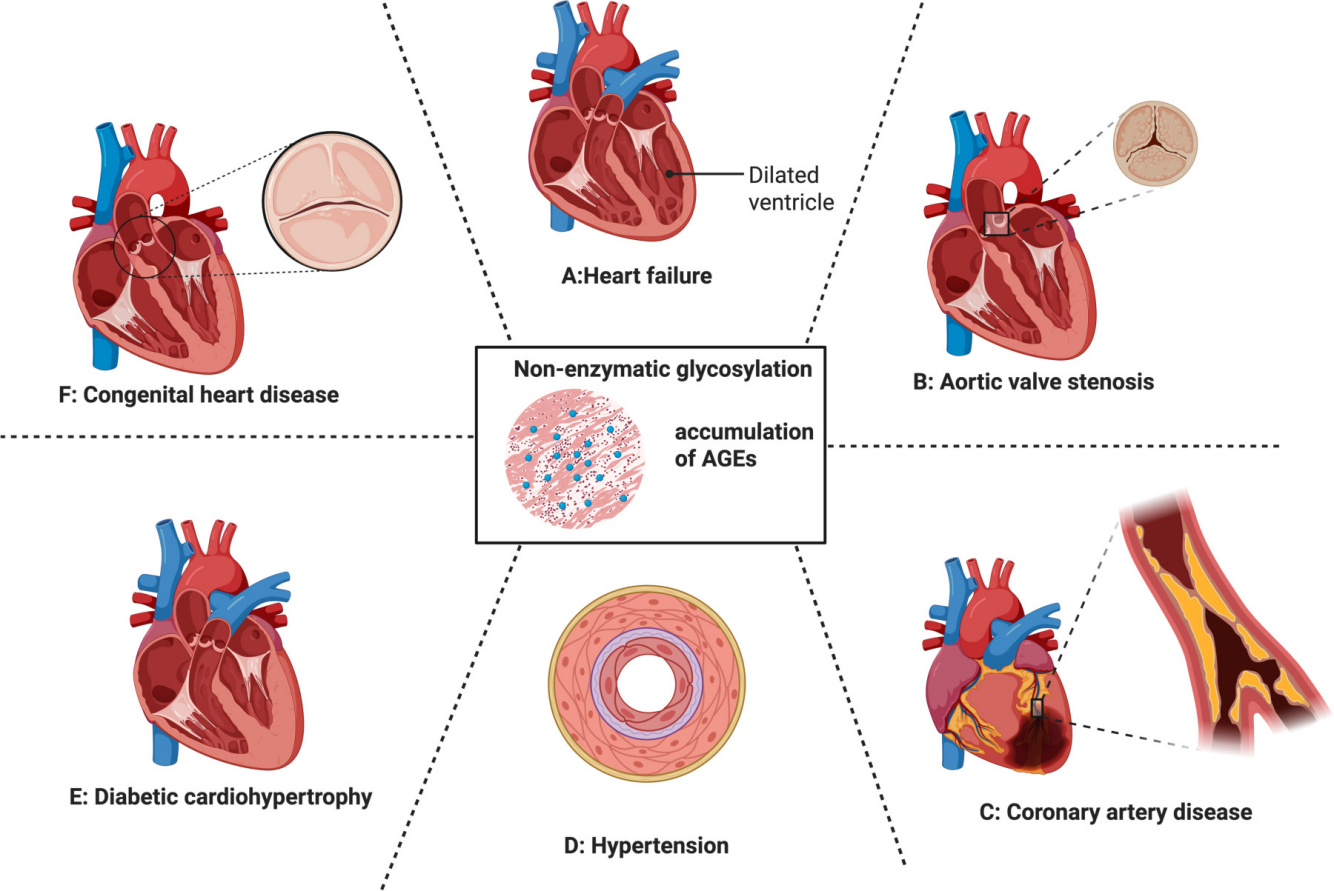
Cardiovascular impact of non-enzymatic glycation and the accumulation of AGEs. From **(A–F)** heart failure, valvular heart disease, coronary artery disease, hypertension, diabetic cardio hypertrophy, congenital heart disease. Each of them were discussed in 5.1–5.6.

The accumulation of AGEs is a key promotive factor for a range of CVDs (Figure 3). The manifestations of the above-mentioned mechanisms of AGE formation in some specific CVD types will be described separately as follows.

### 5.1 AGEs and heart failure

Heart failure is a progressive clinical syndrome resulting from diverse cardiovascular abnormalities, such as coronary artery disease, myocarditis, hypertension, and cardiomyopathy, ultimately leading to impaired atrial or ventricular ejection of blood (131). Plentiful evidence acknowledged that AGEs is related to pathophysiological mechanisms of heart failure (11). Both the accumulation of AGEs in the ECM and activation of AGE-RAGE signaling affects cardiac dysfunction by two central mechanisms (132–134).

Extracellular matrix (ECM) remodeling is a hallmark of heart failure, where pressure overload promotes excessive ECM synthesis, contributing to both diastolic and systolic dysfunction (135, 136). AGE formation manipulates ECM remodeling by altering their structures and functions, especially collagen. AGEs in collagen leads to crosslinks in ECM that contribute to myocardial stiffness and left ventricular ejection fraction (LVEF) (137, 138). In mouse models of heart failure, a reduction in collagen type III alongside the accumulation of advanced glycation end products (AGEs) has been observed. Treatment with crosslink breakers was shown to restore collagen function while simultaneously reducing AGE formation (110). As the most critical AGE precursor, Methylglyoxal (MG) overexpression in transgenic mice increased ECM remodeling in scarring myocardium (139). In the study, Methylglyoxal (MG) was observed with co-localization of collagen I in cardiac tissues after myocardial infarction, suggesting non-enzymatic glycation in cardiac tissue was linked to increased fibrosis and hypertrophy. From a clinical perspective, Willemsen et al. demonstrated that patients with heart failure exhibit elevated tissue levels of AGEs, which are associated with diastolic dysfunction and reduced aerobic exercise capacity (140). While increased AGEs were independently associated with reduced exercise capacity, another study found that either continuous or interval exercise training regimens prevented artery stiffness via mitigation of AGE level (141). Collectively, these studies emphasized the adverse effect of AGEs on heart failure and implied possible therapeutic options of exercises for heart failure patients. AGEs exert their effects mainly by binding to RAGEs and initiating several signaling transduction in the cardiac tissue (142, 143). For example, activation of RAGE enhanced NF-*κ*B activation and promoted myofibroblast transition and oxidative stress in heart failure (144, 145). TGF-β1/Smad pathway was another downstream signaling cascade of the AGE-RAGE axis in myocardial fibrosis, an essential heart failure process. Pretreatment of anti-RAGE antibody substantially decreased TGF-β1 and p-smad2/3 expression of cardiac fibroblasts, suggesting the presence of RAGE/TGF-β signaling pathway in heart failure (146).

### 5.2 AGEs and valvular heart disease

The four major valves in the heart includes three kinds of valvular problems: atresia (block of the valve), regurgitation (backflow of blood through the valve), and stenosis (narrowness of the valve) (147–149). Chronic glycation of proteins has been in many valvular heart diseases, such as stenotic aortic valves, mitral valve disease, and congenital valvular disease (bicuspid aortic valve). By contrast, the accumulation of AGEs is related to disease severity and progression (150–152).

Aortic valve stenosis (AVS), the most common aortic valve disorder, is driven by fibrocalcific remodeling of valve leaflets. Chronic inflammation contributes to calcification (153), and AGE–RAGE signaling promotes valve interstitial cell trans differentiation into osteoblast-like cells with immune cell infiltration (154). A diet rich in carbohydrates and fats enhances AGE–LDL formation, which in turn promotes aortic valve lesions in hamsters. In human aortic valve interstitial cells, AGE–LDL induces the expression of inflammatory mediators, including ICAM-1, IL-6, and ALP, and facilitates calcific nodule formation. Conversely, silencing the RAGE gene attenuates these effects by suppressing NF-κB signaling (155). The sex difference of AGE synthesis was reported in SD rat models, and male SD rats have higher AGEs than female rats, indicating that males are more likely to suffer from CVDs (156). In a study of patients with kidney failure, male patients are more prone to aortic valve calcification with higher AGE accumulation (157).

Heart valve replacement is the only treatment for symptomatic severe valve diseases (158). However, the increasing prevalence of valve degeneration is expected to emerge as a leading cause of cardiovascular morbidity and a significant healthcare burden in the coming decades (159). Several causes contribute to valve degeneration: calcification of the bioprosthetic valve, inflammatory reactions, tissue thickening, and collagen network (160). Immunohistochemistry on 45 clinical explants revealed that AGE and HSA accumulation are standard features in failed BHVs, exhibiting collagen crosslink regardless of calcification severity. Moreover, *in vitro* infiltration of bovine pericardium demonstrated that this effect of AGEs accumulates mainly in lysine and arginine. The process occurs as early as implantation starts, that affects the effective orifice area of the bioprosthetic valve, indicating AGEs as one cause of implantation failure (161).

### 5.3 AGEs and coronary artery disease

Coronary Artery Disease (CAD) is a chronic condition characterized by the progressive narrowing or blockage of the coronary arteries, leading to a complex interplay of endothelial dysfunction, foam cell accumulation, inflammation, and thrombosis (162). Two RNA-binding proteins, nuclear factor 90 (NF90) and nuclear factor 110 (NF110), mediate AGE accumulation in vascular smooth muscle cells (VSMCs) via stimulating the degradation of AGE receptor 1 (AGER1) (163). Atherosclerosis is initiated when LDL becomes retained within the sub-endothelial layer of medium and large arteries. This upregulation of AGE-RAGE cascade in macrophages facilitates increased binding and internalization of modified low-density lipoproteins (LDL), such as oxidized LDL (ox-LDL), into the macrophages. Internalized cholesterol forms cholesterol esters in the macrophage stored as lipid droplets, contributing to foam cell formation in the early stage of atherosclerotic lesions (164). LDL cross endothelial cells (ECs) via transcellular transcytosis, which is mainly mediated by Caveolin-1 protein (165, 166). Interaction between AGE and RAGE on the ECs robustly upregulated Caveolin-1. AGE-RAGE signaling promoted phosphorylation of transcription factor NF-κB, which, in turn, translocated into the nucleus and increased expression of upstream RAGE and Caveolin-1. Therefore, AGE-RAGE-NF-κB forms a positive feedback loop in the LDL transcytosis (117).

As one of the most prevalent and studied AGEs, CML arising through the nonenzymatic glycation and oxidative modification of monosaccharides (such as glucose) and protein-bound lysine residues (167). Notably, *in vivo* experiments suggested that CML accelerates the formation of atherosclerosis via the migration of foam cells into regional lymph nodes. Injection of exogenous CML into diabetic apoE−/− mice promoted plaque formation and cholesterol crystal deposition in the plaques. Moreover, CML upregulates the expression of CD36, a signal molecule expressed by macrophage-derived foam cells, indicating the accumulation of foam cells in atherosclerosis formation. *in vitro* analysis demonstrated that CML inhibited foam cell migration via ROS generation and FAK signaling, which are responsible for macrophage migration (167). A comparative study assesses CML levels as a predictor factor for coronary artery disease in diabetic patients (168). The result showed that patients with more severe coronary artery diseases have higher CML accumulation. Additionally, CML is related to several inflammatory factors up-regulation, such as TNF-α and IL-6, which is highly correlated with early atherosclerotic lesion formation and increased long-term cardiovascular mortality (169).

### 5.4 AGEs and hypertension

Systemic arterial hypertension, commonly referred to as hypertension, is a significant CVD characterized by persistently elevated pressure within the arteries (170). Arterial stiffness is a leading factor in the development of hypertension. Elastic arteries enhance the effects of cardiac contraction during the diastolic stage by storing potential energy when they expand elastically during the systolic stage. However, they become less able to absorb the force of blood ejected from the heart as artery stiffen, leading to higher systolic blood pressure and an increased pulse pressure (171).

AGEs in cardiomyocytes is related to arterial stiffness, demonstrating that higher pulse wave velocity (PWV) and pulse pressure were positively related to elevated plasma AGEs (172). Aging is markedly associated with arterial stiffness due to structural and functional changes in the vascular system (173). One comparative study showed that older participants exhibited a notable increase in PWV with higher plasma AGE levels in an age-dependent manner (174). The relationship between arterial stiffness and AGEs is restricted to patients with vascular diseases and induces early vascular changes before the disease. In men and younger individuals, vascular stiffness was significantly associated with AGE accumulation, implying it as a non-invasive marker for arterial stiffness (175).

Considering the potential involvement of AGEs in arterial stiffness, it would be anticipated that elevated AGE levels correlate with hypertension. Recently, a study investigated the role of elevated AGE levels and CVD in an elderly cohort; however, In the survey, AGEs demonstrated no correlation with hypertension in the cohort, and diastolic blood pressure was even negatively associated with skin autofluorescence (AGE markers) (15). This debate may be explained by age difference in diastolic blood pressure: earlier studies conducted in all age ranges demonstrated that AGE levels were positively related to blood pressure in men and children (176).

Hypertension substantially elevates the risk of mortality associated with cardiovascular events (177). A large-scale meta-analysis examined the association between hypertension and mortality across 67 distinct causes of death. The findings revealed that hypertension was associated with an increased risk of all-cause mortality and elevated risks for 17 specific causes, predominantly circulatory diseases. HGI is a measure that quantifies the difference between observed and predicted HbA1c levels based on blood glucose concentration, reflecting individual variability in of hemoglobin (178). Notably, cardiovascular events in female hypertensive patients increased with higher HGI levels, demonstrating a J-shaped relationship (14).

### 5.5 AGEs and diabetic heart disease

In the past decades, diabetic-induced heart failure, cardiomyopathy, hypertension, and coronary diseases have received markedly research attention to relative mechanisms (179). People with diabetes have two sources of AGE initiation: exogenous and endogenous sources (180). Diabetic cardiomyopathy (DCM) is a distinct pathological condition in which heart failure develops independently of coronary artery disease, hypertension, or valvular abnormalities. It typically manifests first as diastolic dysfunction and progresses over time to heart failure with reduced ejection fraction. In diabetic patients, insulin resistance, compensatory hyperinsulinemia, and persistent hyperglycemia collectively contribute to the heightened susceptibility to DCM (181).

AGEs are generated in DCM development through promoting ferroptosis: AGEs were found to induce ferroptosis in cardiomyocytes via alteration in the expression of key ferroptosis markers, such as Ptgs2, ferritin, and SLC7A11, which in turn, contributing to the development of DCM. Also, AGE increases oxidative stress in the cell via activation of AMPK signaling, aggravating cell viability in DCM mice. These changes further led to cardiomyocyte dysfunction and structural remodeling. Furthermore, the study found that activating the AMPK/NRF2 signaling by sulforaphane attenuates ferroptosis in the AGE-treated group, allowing cardiomyocyte survival and providing potential therapeutic options to activate NRF2 in DCM treatment (182). AGEs bound to human serum albumin in cardiac cells downregulated Nrf-2 expression and its target antioxidant genes Keap1, HMOX1, and NQO1 expression, reducing cellular defense against oxidative damage (183).

Microtubule (MT) dysfunction was identified as a driver for contractile ventricular diseases (184). Increased stability of cardiac microtubules plays a significant role in the cardiac dysfunction observed in STZ-induced type 1 diabetic rats (185). Excessive AGEs cultured cardiomyocytes reduced SIRT2 expression, a gene responsible for cytoskeleton stability and the deacetylation of microtubules in the progression of diabetic cardiomyopathy (DCM). Injection of AGE neonatal rat ventricular myocytes enhances leakage of Ca2 + from the sarcoplasmic reticulum (SR), leading to partial depletion of SR Ca^2+^ stores and a subsequent reduction in systolic Ca^2+^ transients, finally causing contractile dysfunction in diabetic cardiomyopathy (186).

Diabetes is universally acknowledged as an inflammatory disorder, as the impairment in insulin secretion and sensitivity is attributed to factors such as proinflammatory proteins and immune infiltration in DCM patients (187). Previous experimental evidence found that myeloid differentiation 2 (MD2), a co-receptor for toll-like receptor 4 (TLR4), could induce inflammatory response in DCM development. AGEs and MD2-TLR4 form a complex that upregulates MAPK/ NF-*κ*B and other proinflammatory factors (188). Macrophage polarization is crucial in immune responses, inflammation, and tissue remodeling. This plasticity allows macrophages to switch between two central polarization states, M1 (proinflammatory) and M2 (anti-inflammatory or tissue-repairing) (189). Excessive AGE accumulation induces the expression of microRNA miR-471-3p in bone marrow–derived macrophages (BMDMs) from diabetic mice as well as in RAW264.7 macrophage cells. Mechanistically, miR-471-3p directly binds to silent information regulator 1 (SIRT1), which was reported to have an anti-inflammatory role in switching macrophage polarization from M1 to M2 in DCM development (190).

### 5.6 AGEs and congenital heart diseases

AGEs may exert detrimental effects on congenital heart malformations starting from embryo development. For example, the accumulation of CML and MG upregulates smad proteins phosphorylation, thus leading to more borderer myocardium in the cushions of the outflow tract and more likely to develop congenital heart diseases (191). The bicuspid aortic valve (BAV) is the most prevalent congenital heart abnormality and is often linked to abnormalities in the proximal aorta (192). BAV encompasses a range of morphological abnormalities where the aortic valve consists of two functional cusps and has fewer than three distinct zones of parallel cusp apposition (193). One study investigated the lifetime health outcomes of individuals with BAV over a median follow-up of 19.1 years. It showed that a significant portion of deaths in BAV patients were linked to cardiovascular complications, particularly aneurysm formation, aortic dilatation and aortic dissection or rupture (192). BAV patients possess higher levels of AGEs that play a crucial role in cell apoptosis, thus increasing the risk of structural changes in the development of aortic aneurysms and the likelihood of acute aortic dissection. Therefore, the sRAGE may serve as a tool to predict the risk of BAV and BAV-associated aortopathies (194). Mechanistically, higher sRAGE levels correlate with increased activation of the NF-κB pathway, a key regulator of inflammation and cellular stress responses. This activation may contribute to the structural remodeling of aortic aneurysms in BAV patients (195). Increased levels of AGE also enhanced vimentin expression, which is a 48 kDa fragment cleaved by caspase-3 in cell apoptosis. Additionally, vimentin may function as a mechanotransducive regulator of Notch signaling, contributing to arterial wall remodeling (150).

## 6 Therapeutic strategies for CVDs targeting AGE production

**Figure 4:**
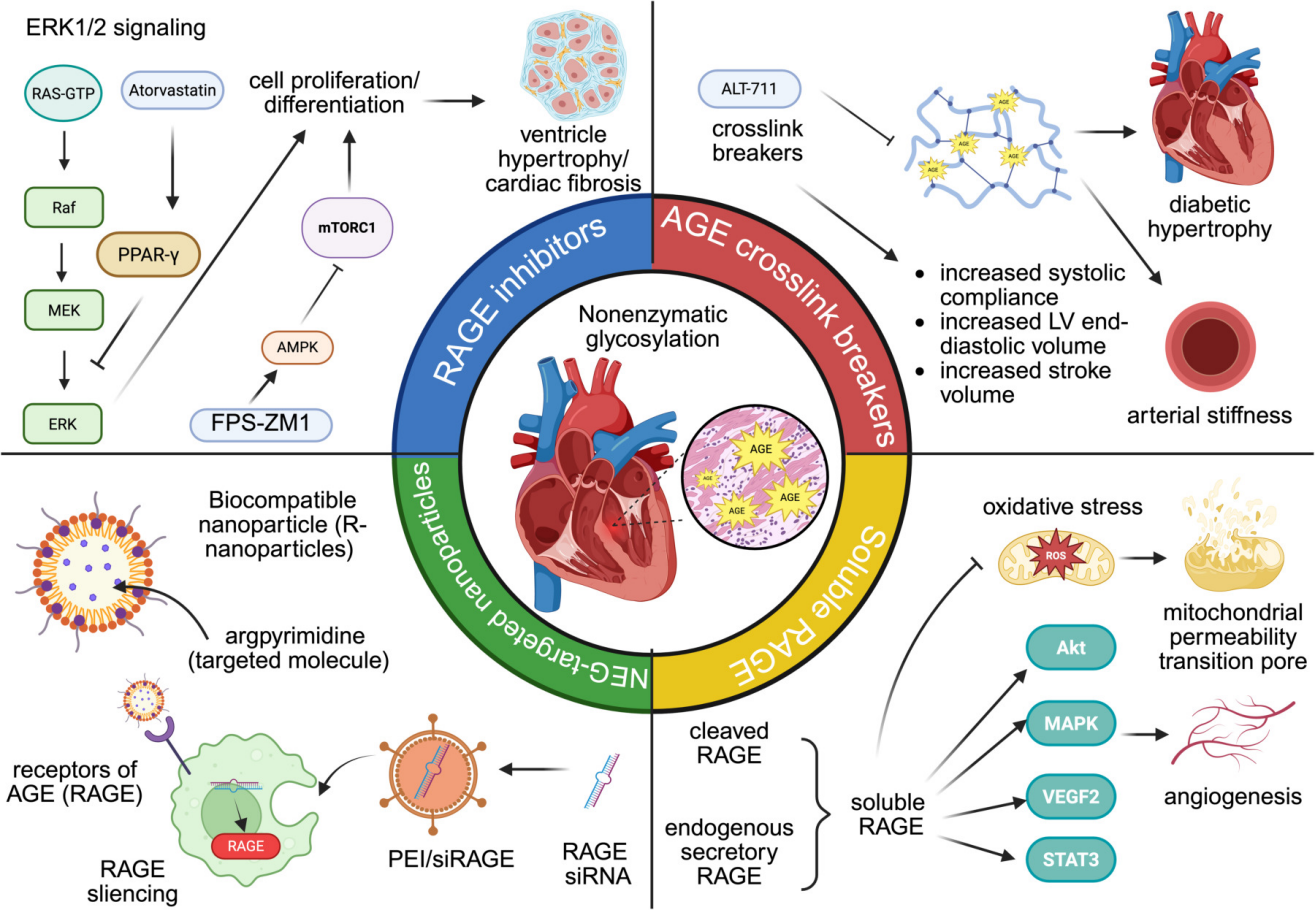
The role of AGE-targeted therapy in cardiovascular diseases. From left to right: RAGE inhibitors (atorvastatin and FPS-ZM1), AGE crosslink breakers (ALT-711), AGE-targeted nanoparticles (R-nanoparticles and PEI/siRAGE), soluble RAGE.

In recent years, experimental evidence supported that target AGEs could offer therapeutic options for patients with CVDs (Figure 4).

### 6.1 RAGE inhibitors

Inhibitors of RAGE can reduce cardiac fibrosis and enhance diastolic function by downregulating AGE-RAGE signaling. Atorvastatin (Ator), originally identified for its lipid-lowering properties, has also been reported to activate peroxisome proliferator-activated receptor gamma (PPAR-γ) signaling. PPAR-γ could exert inhibiting effects on the AGE-RAGE axis, leading to the downregulation of ERK1/2 phosphorylation in rat fibroblast. Previous studies have proved the effects ERK1/2 in cell proliferation in many CVD models, such as left ventricle hypertrophy and cardiac fibrosis (196). Similarly, FPS-ZM1, another RAGE-specific inhibitor, upregulates AMPK phosphorylation and downregulates mTOR/NF-κB in cardiac tissue. Activation of AMPK mediates protective effects against systolic overload-induced cardiac dysfunction, whereas activation of mTOR/ NF-κB has detrimental effects that act as pro-inflammation and pro-apoptotic drivers. Consequently, the blocking effects of FPS-ZM1 suppressed inflammation and cardiac remodeling in heart failure (197). Although RAGE inhibition holds promising therapeutic potentials in AGE-mediated cardiovascular injury, several safety concerns in the application of RAGE inhibitors in other diseases warrant more investigation in future clinical trials. In early Phase I and II trials of Alzheimer disease, high doses of azeliragon (small-molecule RAGE antagonist) could increase falls, confusion, and cognitive decline, leading to early discontinuation of the high-dose arm (198).

### 6.2 AGE crosslink breaker

AGE-crosslink breakers restore cardiovascular function by disturbing the formation of glycogen-dependent crosslinks. ALT-711 is a phenyl-4,5-dimethyl thiazolium chloride derivative used in many types of CVDs, including systolic hypertension, heart failure, and other aging-related heart dysfunction (199, 200). Mechanistically, ALT-711 effectively reverses significant artery stiffness in diabetic animals, restoring vascular properties toward normal levels. Diabetic mice treated with ALT-117 for three weeks demonstrated increased systemic arterial compliance (0.87 ± 0.08 ml/mmHg than 0.56 ± 0.05 ml/mmHg in untreated diabetic mice). Similarly, carotid artery compliance rose from 0.28 ± 0.03 mm^2^/mmHg in untreated diabetics to 0.45 ± 0.05 mm^2^/mmHg post-treatment (201). Another study investigated the effect of ALT-711 on left ventricle stiffness. After one month of daily ALT-711 administration, a significant reduction of approximately 40% in LV stiffness was observed, with measurements decreasing to 33.1 ± 4.6 mm Hg·m^2^/ml. This decrease in stiffness was accompanied by improvements in cardiac function, including increased LV end-diastolic volume and stroke volume, without significant changes in systolic blood pressure or heart rate (202).

### 6.3 Soluble RAGE

Soluble forms of RAGE (sRAGE) could be generated from two primary sources: first, cleaved RAGE (cRAGE) is generated through the proteolytic shedding of membrane-bound RAGE by matrix metalloproteinases; second, endogenous secretory RAGE (esRAGE) is synthesized from RAGE gene. sRAGE has protective effects for cardiomyocytes via suppression of cell apoptosis and mitigation of angiogenesis, making it one of the hot points of therapy strategy in CVDs. sRAGE significantly restored left ventricular end-diastolic volume (LVEDV) and left ventricular end-systolic volume (LVESV) in I/R mice. Immunohistochemical staining for CD31 revealed increased capillary density in sRAGE-treated hearts, suggesting that sRAGE promotes angiogenesis following I/R injury. Meanwhile, the expression of pro-angiogenic genes, such as Akt, MAPK signals, VEGFR2, and STAT3, were significantly increased in sRAGE-treated cardiac microvascular endothelial cells (CMECs). Collectively, these angiogenic effects lead to improved cardiac function and reduced tissue damage post-injury (203). sRAGE treatment could also downregulate the expression of apoptosis markers and autophagy-related proteins. Therefore, I/R injured myocardial tissues observed reduced TUNEL-positive cells, lower caspase-3 activity, and decreased autophagosome formation (204). Cardiac dysfunction can lead to the opening of the mitochondrial permeability transition pore (mPTP), subsequent generation of reactive oxygen species, and mitochondrial calcium overload. Exogenous administration of sRAGE inhibits mPTP opening and mitochondria-mediated apoptosis by suppression of mitochondria cytochrome c, caspase 3, and caspase nine release (205).

### 6.4 Nanoparticles that target AGEs

In recent years, nanoparticles (NP) have been developed as an innovative drug delivery system, utilizing 1–100 nm biomaterials to transport therapeutic agents into diseased tissues (206). NPs targeting the AGE accumulation are an emerging class of agents for therapeutic interventions and diagnostic imaging in other diseases. For example, one study constructs a novel biocompatible nanoparticle encapsulated with argpyrimidine, an AGE that targets the nanoparticle to cell surfaces that express RAGE. The results showed that compared to free soluble anti-hyperglycemia drugs, anti-hyperglycemia drugs encapsulated inside argpyrimidine are more effective in controlling hyperglycemia in diabetic rats. Moreover, rats treated with nanoparticles demonstrated better structural integrity in heart integrity and less cardiac fibrosis formation (207). As discussed above, AGE/RAGE is associated with inflammation status in ischemia injury. PEI/siRAGE is a small interfering RNA nanoparticle that selected RAGE as the target gene to assess its *in vivo* efficacy. Compared with a control group treated with saline, PEI/siRAGE reduces more than 10% of the infarcted size in cardiac tissue, exhibiting a beneficial effect against myocardial infarction (208). Curcumin and aged garlic extract are modified herbal medicines that demonstrated anti-oxidative and anti-glycation impacts in treating CVDs (209, 210). However, limited solubility and low bioavailability impede their efficacy for more cardiovascular applications. A study investigated the impact of nanomedicine based on these two herbal medicines targets both AGEs and ROS in streptozotocin-induced diabetic rats: Both nano-curcumin and AGE demonstrated downregulation of RAGE gene level, and histopathological results also exhibited higher structural integrity in DCM tissue (211). The study by Lu et al. reveals a novel advantage of engineered macrophage membrane-coated siRNA nanoparticle (MMM/RNA NP) platform with overexpression of RAGE in the surface of MMM/RNA NP, through which the nanoparticle effectively achieved higher cellular internalization to the site of myocardial ischemia–reperfusion injury, leading to improved left ventricular ejection fraction (LVEF) and fractional shortening (LVFS) in ischemic mice (212).

## 7 Clinical translation of AGE targeted therapy

Over the past decades, extensive preclinical evaluations of various AGE-derived therapeutics have demonstrated their impressive potential for clinical use. The first clinical trial that demonstrated the feasibility of AGE-targeted therapy recruited 93 participants with elevated pulse pressures were assigned to receive either 210 mg of ALT-711 or a placebo daily for 56 days. The results demonstrated that ALT-711 significantly improved total arterial compliance (15% vs. no change) and reduced pulse pressure (−5.3 vs. −0.6 mm Hg) compared to the placebo group (213). In recent years, 11 other clinical trials of ALT-711 under FDA approvals for treating CVDs have been registered (https://clinicaltrials.gov/) (Table 2). Although the elimination of AGE in CVDs holds a promising concept, few documented trials show that AGE-targeted therapy is more favorable than other standard therapy (214). For example, synthetic AGE inhibitors that reached phase I/II clinical trials such as aminoguanidine or alagebrium both faced toxicity issues including major liver/kidney toxicity and autoimmune-like reactions (215). In recent years, nature products have been evaluated for their antiglycation activity. Gallic acid (GA) is a plant-derived polyphenol present in gallnuts, grapes, tea leaves, and oak bark with antioxidant activities. Treatment of GA in cardiac H9C2 (2-1) cells decrease intracellular ROS and oxidant level (*P* < 0.05), suggesting the cytoprotection efficacy of GA (216). Epigallocatechin-3-gallate (EGCG) is a major catechin found predominantly in green tea, valued for its potent antioxidant, anti-inflammatory, and potential disease-preventive properties. In simulated glycation systems, EGCG inhibited the formation of AGEs by 54.44% and α-dicarbonyl compounds (key intermediates of AGEs) by 84.47%, which is attributed to free radical scavenging and stabilization of protein secondary structure (217). Kaempferol, a polyphenol derived from green tea, has been shown to downregulate RAGE expression and its downstream effector NF-κB p65, thereby suppressing glycation-induced inflammatory signaling. This anti-inflammatory effect mitigates myocardial injury by reducing oxidative stress, apoptosis, and fibrosis, particularly under diabetic conditions where AGE–RAGE activation sustains chronic inflammation. Histological and ultrastructural evidence further supports its cardioprotective role, demonstrating preserved mitochondrial integrity and intact myofibril architecture during ischemia–reperfusion injury (218). Nevertheless, most natural compound of AGE inhibitor/crosslink breakers were still in pre-clinical stage and future clinical studies are required to validate their efficacy in CVD diseases.

**Table 2:** FDA-approved clinical trials of AGE-targeted therapy.

**ClinicalTrials.gov** **ID**	**Current status**	**Study design**	**Therapeutic use**	**Enrollment**
NCT01913301	Terminated	Phase 2	Diastolic heart failure	134
NCT01417663	Completed	Phase 2/phase 3	Restored cardiovascular structure and function	48
NCT01014572	Completed	Phase 2	Cardiovascular restoration in healthy older individuals	62
NCT00739687	Terminated	Phase 2	Patients with chronic heart failure	100
NCT00662116	Terminated	Phase 2	Influence of ALT-711 treatment on exercise capacity in individuals with diastolic heart failure	160
NCT00516646	Completed	Phase 2	Evaluation of ALT-711 in individuals with chronic heart failure	100
NCT00302250	Completed	Phase 2	Relationship between vascular distensibility and endothelial-dependent vasoreactivity in systolic hypertension patients treated with ALT-711 or placebo	70
NCT00277875	Completed	Phase 2	Potential of ALT-711 to improve vascular function by breaking AGE	25
NCT00089713	Terminated	Phase 2	Effect of ALT-711 in combination with hydrochlorothiazide in hypertension patients	392
NCT00045994	Completed	Phase 2	Efficacy of ALT-711 to isolated systolic hypertension	180
NCT00045981	Completed	Phase 2	The effect of ALT-711 to systolic hypertension in patients without left ventricular hypertrophy	450

From clinicaltrials.gov, accessed 06-04-2025.

In addition to its therapeutic uses, AGE level is also utilized as a prognostic indicator in cardiovascular diseases (CVDs). HbA1c is produced when glucose non-enzymatically and irreversibly attaches to the N-terminal valine of the β-chain of hemoglobin in red blood cells (219). HGI represents individual variations in the correlation between plasma glucose and HbA1c and was initially employed to diagnose diabetes mellitus. Furthermore, several studies have associated high HGI levels with significant diabetes mellitus complications, including CVD (220). One study examined the link between cardiovascular risk in diabetic patients and elevated HGI. Patients with varying HGI levels experienced different outcomes following intensive treatment: both the low and moderate HGI groups (low HGI ≤ −0.520 and moderate −0.520 to 0.202) showed a reduction in MI, stroke, or death from cardiovascular causes. Conversely, high HGI was not linked to treatment outcomes and even increased the overall mortality rate by 41% (221). Exploring the connection between HGI and cardiovascular outcomes reveals a U-shaped relationship, where both low and high HGI levels are associated with heightened risks. Lin et al. concentrated on patients with diabetes and coronary artery diseases, discovering that both low and high HGI levels were tied to increased MACE risks, with low HGI significantly raising the risk of cardiovascular death (222). Among 5,260 patients with coronary artery diseases, high HGI was linked to increased 365-day mortality, while low HGI was associated with 30-day mortality, indicating HGI's correlation with both short-term and long-term mortality in the progression of coronary artery disease (220). Cardiovascular risks and elevated HGI were also suggested in heart failure (223, 224) and myocardial injury (225).

One significant drawback of using the HGI as a prognostic tool is that numerous studies concentrate solely on CVD patients who have diabetes. The scarcity of research on HGI in non-diabetic individuals might undermine the unique pathophysiological roles of AGEs could have in diabetic vs. non-diabetic CVD cases. For instance, a study identified a linear association between HGI levels and the severity of CAD in non-diabetic people. In CAD patients, HbA1c levels rose progressively with the severity of clinical symptoms, with stable angina patients, unstable angina patients, and those with acute myocardial infarction exhibiting the lowest, intermediate, and highest HGI levels, respectively (226). Conversely, another study examined the link between HbA1c levels and both all-cause and CVD mortality in elderly non-diabetic individuals, finding that both low and high HbA1c levels are linked to heightened risks of all-cause mortality in older patients (227). Future studies should investigate the role of HGI in CVDs among both diabetic and non-diabetic groups to gain a better understanding of HGI's function and improve its effectiveness in clinical risk evaluation.

## 8 Discussion

As the key metabolic byproducts in non-enzymatic glycation, Advanced glycation products (AGEs) can lead to extensive physiological and pathological consequences. The association between AGEs and cardiovascular pathology is well-documented, especially in relation to diabetes mellitus and metabolic syndrome. Elevated glucose levels accelerate the production of AGEs, which irreversibly modify structural proteins and functional proteins in cardiovascular tissues (228, 229).

AGEs primarily exert their harmful effects by engaging the AGE-RAGE axis, a well-known signaling pathway that initiates OS and persistent inflammation (230). When AGEs bind to the RAGE, it sets off a series of intracellular signaling pathways, such as NF-κB, JAK-STAT, MAPK, and PI3K/AKT, which intensify OS and inflammatory reactions (95). In addition to activating proinflammatory signaling, AGEs directly impair cardiovascular cell populations by inhibiting autophagy, inducing apoptosis, disrupting endothelial repair through endothelial progenitor cells (EPCs), and promoting fibrosis via myofibroblast transformation. Moreover, AGE modification of plasma proteins alters lipid metabolism, increasing the atherogenic potential of lipoproteins and thereby accelerating plaque development.

Although oxidative stress (OS), chronic inflammation, and the modification of structural proteins are common mechanisms driving the pathological effects of AGEs, the specific consequences differ based on the cardiovascular condition, such as heart failure, valvular heart disease, coronary heart disease, systolic hypertension, and congenital heart disease. In the context of diabetes mellitus, persistent high blood sugar levels hasten non-enzymatic glycation, a natural reaction between reducing sugars and biological macromolecules like proteins, lipids, and nucleic acids (231). This prolonged hyperglycemia speeds up the production of AGEs from both internal and dietary origins (232). The clinical significance of AGEs has shown that AGE-targeted therapy can slow the progression of CVDs. Apart from drug intervention, emerging evidence suggests diet can modulate the systemic AGE burden and thereby potentiate pharmacologic or nutraceutical AGE-targeted strategies. Several small clinical trials report that restricting dietary AGE intake, even over short to moderate periods, significantly lowers circulating AGE levels and reduces inflammation and oxidative stress in both healthy individuals and those with cardio-kidney-metabolic (CKM) conditions (233). Furthermore, utilizing the hemoglobin glycation index effectively predicts the onset of CVD in both diabetic and non-diabetic individuals (234).

Despite encouraging outcomes in the investigation of AGEs and its therapeutic applications, several significant challenges hinder their complete understanding, clinical translation, and therapeutic targeting (Table 3). In recent years, the development of precision medicine also suggested heterogeneity of AGE level in different cohorts. Therefore, updates in multi-omics association studies may help to identify AGE-targeted treatment options. Adams et al. measured AGE levels in 506 individuals and heritability (h^2^) of serum AGEs was estimated at 0.628, revealing a substantial heritable component for serum AGE levels. Likewise, the DHS MIND Study reported 8 single nucleotide polymorphisms in the genetic component of AGEs, which have been related to higher serum AGE levels (235). One study collected data from 4,182 individuals in the Long-Life Family Study that identified 17 gene expressions (Bonferroni *p* < 2.73 × 10^−^⁶) pleiotropically linked to the eGFR (a measure of kidney function) and sRAGE in kidney aging. These transcriptomic investigations highlight gene-expression architecture in AGE expression suggesting further investigation of AGE variants and heart diseases (236).

**Table 3:** The list of current challenges and further research attention of non-enzymatic glycation.

**Challenges**	**Future research focus**
• **Complex interplay with oxidative and inflammatory stress** • **Difficulty to quantify and model AGEs *in vivo***	• Non-invasive imaging and biomarkers to track AGE deposition and dynamics. • Develop simplified assays for early detection of AGE burden in clinical settings
Diversity and instability of AGEs	
• **Heterogeneous array of early glycation products and irreversible AGEs**	• Utilize advanced techniques to identify and quantify molecular Profiling of AGEs. • Study the differential accumulation and effects of AGEs in specific cardiovascular tissues
Crosstalk with other pathways	
• **AGEs interacts with numerous signaling cascades—NF-κB, JAK-STAT, MAPK, PI3K/AKT, TLR4 and so on, raising the challenge of pathway specificity when targeting glycation therapeutically**	• Investigate the distinct roles and interactions among AGE receptors (RAGE, AGE-R1/R2/R3, CD36, SR-A, TLRs) in cardiovascular cells
Overlapping pathophysiological mechanisms	
• **Lack of diabetic vs. non-diabetic Stratification cardiovascular patients**	• Differentiation cardiovascular effects of AGE in diabetic and non-diabetic patients to identify disease-specific mechanisms and tailor therapeutic strategies. • Identify patients with different HGI levels to personalize risk prediction and treatment
Limited efficacy of AGE-targeting therapies	
• **Inconsistent efficacy and limited long-term safety data such as ALT-711**	• Clarity on the optimal therapeutic window • Conduct long-term clinical trials to elucidate cardiovascular effects of AGE-targeted therapy

In conclusion, the role of AGEs in cardiovascular pathology is increasingly recognized as a critical mechanistic axis that underlies multiple forms of CVDs. This highlights several promising targets—particularly the inhibition of the AGE–RAGE axis may offer substantial benefits for future CVD treatment.
